# Heteroatom Modification Enhances Corrosion Durability in High‐Mechanical‐Performance Graphene‐Reinforced Aluminum Matrix Composites

**DOI:** 10.1002/advs.202104464

**Published:** 2022-06-15

**Authors:** Yuming Xie, Xiangchen Meng, Yuexin Chang, Dongxin Mao, Zhiwei Qin, Long Wan, Yongxian Huang

**Affiliations:** ^1^ State Key Laboratory of Advanced Welding and Joining Harbin Institute of Technology Harbin 150001 China

**Keywords:** aluminum matrix composites, corrosion, fluorographene, interfaces, microstructures

## Abstract

The antagonism between strength and corrosion resistance in graphene‐reinforced aluminum matrix composites is an inherent challenge to designing reliable structural components. Heteroatom microstructural modification is highly appreciated to conquer the obstacle. Here, a bottom‐up strategy to exploit the heterogeneous phase interface to enable high corrosion durability is proposed. Deformation‐driven metallurgy derived from severe plastic deformation is developed to produce Mg‐alloyed fluorinated graphene structures with homogeneous dispersion. These structures allow for absorbing corrosion products, forming a dense protective layer against corrosion, and local micro‐tuning of the suppression of charge transfer. This results in superior corrosion resistance with an outstanding strength‐ductility balance of the composites via ultrafine‐grained and precipitation strengthening. The anti‐corrosion polarization resistance remains 89% of the initial state after 2‐month immersion in chloride‐containing environment, while the ultra‐tensile strength and elongation of 532 ± 39 MPa and 17.3 ± 1.2% are obtained. The economical strategy of heteroatom modification broadens the horizon for anti‐corrosion engineering in aluminum matrix composites, which is critical for the design of carbonaceous nanomaterial‐reinforced composites to realize desired performances for practical applications.

## Graphical abstract

A bottom‐up strategy is proposed to exploit the heterogeneous phase interface to enable high corrosion durability via deformation‐driven metallurgy. The anti‐corrosion polarization resistance remains 89% of the initial state after 2‐month immersion in chloride‐containing environment, which is critical for the design of carbonaceous nanomaterial‐reinforced composites to realize desired performances for practical applications.

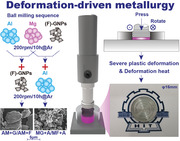

## 1 Introduction

Sparked by its in‐plane impermeability to nearly all molecules,^[^ ^1^ ^]^ graphene and its derivatives are emerging as anti‐corrosion supplementary substances for metals and ceramics.^[^ ^2^ ^]^ This barrier characteristic and chemical stability make it not surprising to shield the metals underneath it from unexpected reactions and corrosion.^[^ ^3^ ^]^ However, when these nanomaterials are applied to prepare aluminum matrix composites in practical applications, especially for high specific‐strength functions, fatal acceleration of the corrosion rate is often to be obtained instead of corrosion improvement.^[^ ^4^ ^]^ The undesirable anti‐corrosion weakening of graphene reinforced aluminum matrix composites can be attributed to three major inherent issues: (a) Graphene can trigger corrosion‐promotion activity (CPA) to enhance the localized galvanic corrosion due to the heterogeneous corrosion potential, such as graphene (0.2 V vs saturated calomel electrode, SCE) and aluminum matrix (−1.1 V vs SCE).^[^ ^5^ ^]^ When coupling with a relatively active metal, the electrochemical stable and high electrically conductive graphene tends to behave like noble cathode, contributing to the formation of micro‐galvanic corrosion that initiates localized corrosion and boosts the anodic dissolution;^[^ ^6^ ^]^ (b) Small molecules such as O_2_ and H_2_O may infiltrate from the edge defects of graphene over the long‐term application, which consequently accelerated the corrosion rate;^[^ ^7^ ^]^ (c) Graphene is easy to agglomerate and exhibits poor compatibility due to its large specific surface area and strong van der Waals interaction, which severely reduces their dispersibility in the aluminum matrices and deteriorates the corrosion resistance performances.^[^ ^8^ ^]^

In light of these, several tailoring strategies were proposed to suppress the CPA behavior toward highly applicable aluminum matrix composites. Heteroatom tailoring of graphene shows great potential for changing the electronic density to tailor its electronic and electrochemical properties.^[^ ^9^ ^]^ It can reduce the electrical conductivity by increasing the electron scattering in graphene lattice, as well as modifying the local electron density to inhibit the electrochemical activity of graphene. Fluorinated graphene is promising to enhance the anti‐corrosion performance for the insulating nature induced by F doping, as well as the molecule impermeability inherited from graphene.^[^ ^10^ ^]^ An alternative to suppressing the corrosion rate is to design microstructures that are intrinsically electrochemically homogeneous (for example, dispersing the second phases through severe plastic deformation engineering^[^ ^11^ ^]^). However, the combination of these two approaches loses effectiveness since the composites reinforced by fluorinated graphene are susceptible to weak interfacial bonding and low load‐bearing capacity due to the chemical inertness of this modified graphene. The desirable interfacial bonding cannot be simply obtained by the high‐value plastic strain induced by severe plastic deformation. Developing cheap but effective microstructure solutions that enable both high corrosion resistance and mechanical performances thus remains a fundamental challenge.

Here we propose a bottom‐up strategy based on the principle of exploiting great interfacial bonding between fluorinated graphene nanoplatelets (F‐GNPs) and aluminum matrices via Mg alloying and severe plastic deformation. A severe plastic deformation technique, deformation‐driven metallurgy (DDM), was applied to obtain the homogeneous dispersion of F‐GNPs to realize extraordinary corrosion resistance. Corrosion‐suppression activity (CSA) induced by the synergistic strategy of active metal and heteroatomic modification was studied. This work aims to examine the effectiveness of synergistic modification and DDM techniques in terms of corrosion behavior in an aggressive chloride‐containing environment. Microstructural factors, interfacial characteristics, reinforcement dispersion, and electrochemical response were evaluated and discussed in detail.

## 2 Results and Discussion

### 2.1 Microstructural Factors

**Figure 1:**
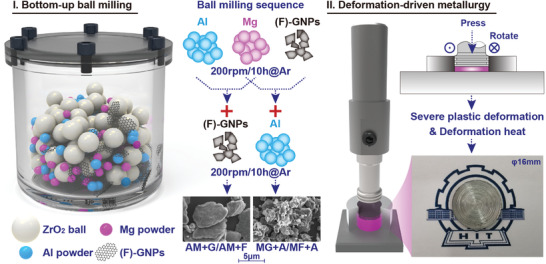
Bottom‐up preparation route of the aluminum matrix composites.

Based on the principle of heteroatom modification toward high corrosion durability, we designed a type of carbonaceous nanomaterial‐reinforced aluminum matrix composites with great interfacial bonding via Mg‐alloyed F‐GNPs and severe plastic deformation. **Figure** **1** depicts the preparation route, including a bottom‐up high‐energy ball milling and a deformation‐driven metallurgy (DDM) process, whose details can be found in Supplementary Information SI‐1. Four kinds of ball milling sequences were selected and denoted as AM+G, AM+F, MG+A, and MF+A. AM+G and AM+F refer to mixing the Al powders and Mg powders firstly and then mixing these blended powders with graphene nanoplatelets (GNPs) and F‐GNPs via ball milling, respectively. MG+A and MF+A refer to mixing the Mg powders and GNPs and F‐GNPs, respectively, and then mixing these blended powders with Al powders. Since the (F)‐GNPs added in the first step separate the Mg powders and Al powders physically and avoid the formation of viscous Al‐Mg intermetallic compounds, the ball‐milled powders of MG+A/MF+A are significantly smaller than those of AM+G/AM+F, indicating that these powders have lower viscosity and tend to be blended homogeneously during the ball milling stage. The morphology of the bulk sample after DDM is shown at the bottom right of Figure 1. Clear concentric circle streamlines can be seen, confirming the existence of the severe plastic deformation process.

**Figure 2:**
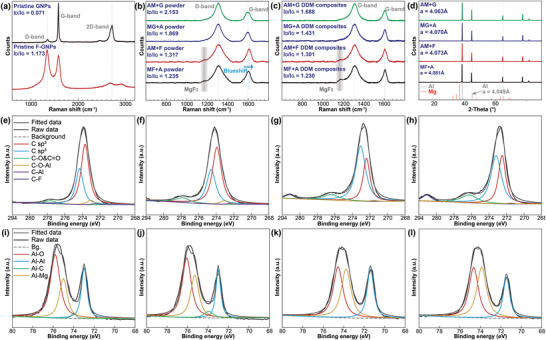
Characterization of the microstructural evolution: a) Raman spectra of the pristine reinforcements, b) Raman spectra of the ball‐milled powders, c) Raman spectra of the DDM composites, and d) XRD results of the DDM composites. XPS results (C 1s and Al 2p) of the DDM composites: e) and i) AM+G, f), and j) MG+A, g) and k) AM+F, h) and l) MF+A.

To access the influence of different bottom‐up preparation routes on the microstructural factors of the (F)‐GNPs and the matrices, we conducted Raman spectroscopy, as shown in **Figure** **2**a–c. The ratio of D‐band to G‐band in the Raman spectrum characterizes the damage degree of graphene structures, which is denoted as *I*_D_/*I*_G_^[^ ^12^ ^]^ A higher *I*_D_/*I*_G_ value refers to the more broken graphene, indicating that there are more active open edges to provide necessary conditions for subsequent metallurgical bonding.^[^ ^13^ ^]^ Compared with the original (F)‐GNPs, an increase in this value can be seen in all the ball milling routes. Due to the more significant lubricating effect of the fluorinated graphene,^[^ ^14^ ^]^ the crushing effect caused by ball milling is weakened in the AM+F and MF+A routes. On the one hand, the integrity of F‐GNPs themselves is better preserved, resulting in a more efficient load transfer effect for mechanical performances and surficial absorbing integrity for corrosion resistance. On the other hand, it also means the reduction of the active reaction sites. It is also worth noting that the characteristic peak of MgF_2_ is observed in Figure 2b,c. Since MgF_2_ has high chemical stability and bond energy,^[^ ^15^ ^]^ the existence of this substance implies the strong chemical bonding between the carbonaceous phases and the matrices. This phenomenon compensates for the adverse effects of fewer active sites during the ball milling process. In addition, the ball milling routes of preferential mixing of (F)‐GNPs in the first step have a low *I*_D_/*I*_G_ value, indicating that the physical isolation effect between Al and Mg brought by (F)‐GNPs can reduce the overreaction of the ball‐milled powders. Figure 2c shows the evolution characteristics of the bulk DDM composites, in which all the *I*_D_/*I*_G_ values have decreased correspondingly. Obviously, graphene cannot be re‐spliced into a larger piece via severe plastic deformation.^[^ ^16^ ^]^ The decrease in this value indicates that the decrease in the thickness and layers of the (F)‐GNPs, and finally results in the increase of the specific surface area of graphene. This provides better prerequisites for the load transfer effect. In addition, the results of X‐ray diffraction (XRD) tests (Figure 2d) show that only the *α*‐Al phase can be seen in all the composite materials. Since the XRD has a concentration detection limit, homogeneously dispersed nanophase with low concentration cannot be detected in the bulk materials. This indicates that there are no large‐sized Mg clusters or undesirable large‐sized Al‐Mg intermetallic compounds in the matrix. In other words, the presence of many nano‐magnesium particles in the matrix provides sufficient reaction sites for the solid‐state alloying between the (F)‐GNPs and the matrices.

X‐ray photoelectron spectroscopy (XPS) was also applied to analyze the microstructural evolution and further illustrate the bonding behavior of the DDM composites. Figure 2e–h shows the deconvolution results of C 1s peak. Compared with the GNP‐reinforced composites, F‐GNP‐reinforced composites have a higher sp^3^ to sp^2^ ratio, which is in line with the bonding form of F‐GNPs, that is, one C atom forms a bond with two in‐plane C atoms and one out‐of‐plane F atom.^[^ ^17^ ^]^ Besides, there are basically no C‐Al bonds in the F‐GNPs DDM composites, which proves that the existence of F atoms isolates the direct reaction between Al matrix and C atoms, thereby avoiding the formation of the easily hydrolyzable compounds Al_4_C_3_. Since this substance is detrimental to the structural integrity of materials exposed to the aqueous environment,^[^ ^18^ ^]^ this phenomenon provides the possibility to improve corrosion resistance. Figure 2i–l depicts the deconvolution results of Al 2p peak. Different ball milling routes do not seem to change the ratio distribution of the chemical bonds, but the binding energy of the composite added with the F element has been significantly reduced, indicating that the number of electrons lost by the Al atom during the metallurgical process is reduced. This implies that Al is less directly involved in the reaction with carbonaceous substance and partially reacts with Mg to form nanophases (Al in Al‐Mg intermetallic compounds tends to gain electrons^[^ ^19^ ^]^). This is consistent with our vision properly, which is to achieve indirect bonding between aluminum and (F)‐GNPs through Mg alloys, thereby avoiding the CPA effect and improving the comprehensive corrosion resistance of the DDM composites.

**Figure 3:**
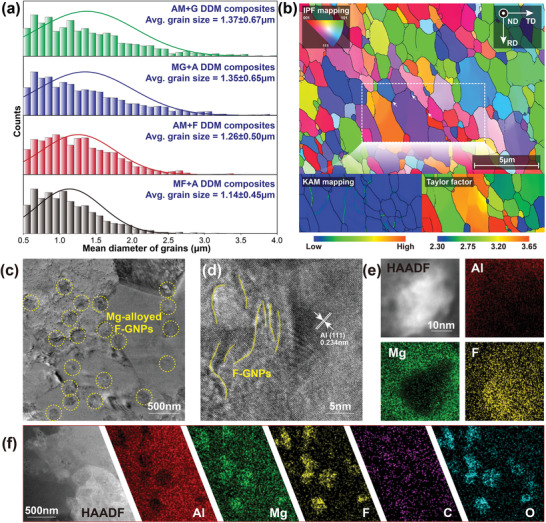
Microstructural characterization images of the DDM composites via EBSD and TEM techniques: a) distribution of the grain sizes, b) grain morphologies of the MF+A DDM composites, c) distribution of the Mg‐alloyed F‐GNPs, d) local phase interface, e) core‐shell structures formed by Mg alloying, and F‐GNPs, and f) EDS characterization of the Mg‐alloyed F‐GNPs.

**Figure** **3**a depicts the grain size distribution of the composites prepared by four bottom‐up routes. More detailed information can be obtained in Supplementary Information SI‐3. This result can be explained from the following two aspects: (1) F‐GNP has an excellent lubricating effect similar to polytetrafluoroethylene,^[^ ^20^ ^]^ which significantly promotes the flow intensity of the composites during the severe plastic deformation process. More obvious dynamic recovery and recrystallization are triggered by the enhanced lattice distortion and defect energy;^[^ ^21^ ^]^ (2) by adding (F)‐GNPs in the first step of the ball milling process, the excessive reaction of Al and Mg can be avoided, thereby reducing the dynamic viscosity of the thermo‐plasticized composites during DDM process. This makes the material more prone to plastic deformation and further improves the grain refinement effect. From the above two explanations, it can be found that the DDM composites obtained via the MF+A route has achieved the finest microstructures with an average grain diameter of 1.14 ± 0.45 µm. We further observed the grain characteristics of the MF+A DDM composites, as shown in Figure 3b. The equiaxed grains occupy the primary component and the residual stress remaining in the microstructures is low to show a limited geometric necessary dislocation density. This implies that the internal defect energy after DDM process is low, thus avoiding the CPA effect caused by the energy release of the internal stress.

Figure 3c shows the transmission electron microscopy (TEM) morphologies of these DDM composites. F‐GNPs are homogeneously dispersed in the matrix, and they have a good lattice matching with the Al matrix (Figure 3d). It indicates good compatibility between the matrix and the reinforcements.^[^ ^22^ ^]^ From the energy spectrum results, the distribution area of Mg and F‐GNPs overlaps, indicating that a self‐assembled nanoscale structure is formed between Mg and graphene. As shown in Figure 3e, the Mg element mainly surrounds the distribution of the F‐GNP particle, presenting a transition structure of the F‐GNP core to the Mg shell to the Al matrix. We call this nanoscale structure Mg‐alloyed F‐GNPs. It avoids the formation of detrimental intermetallic compounds Al_4_C_3_, and enhances the bonding strength between the Al matrix and the reinforcements, since F‐GNPs are relatively chemically inert to aluminum.^[^ ^23^ ^]^ In addition, due to the poor electronic conductivity of the fluoride,^[^ ^24^ ^]^ it also provides a necessary condition for reducing the CPA effect on the composites.

### 2.2 Mechanical Performances

**Figure 4:**
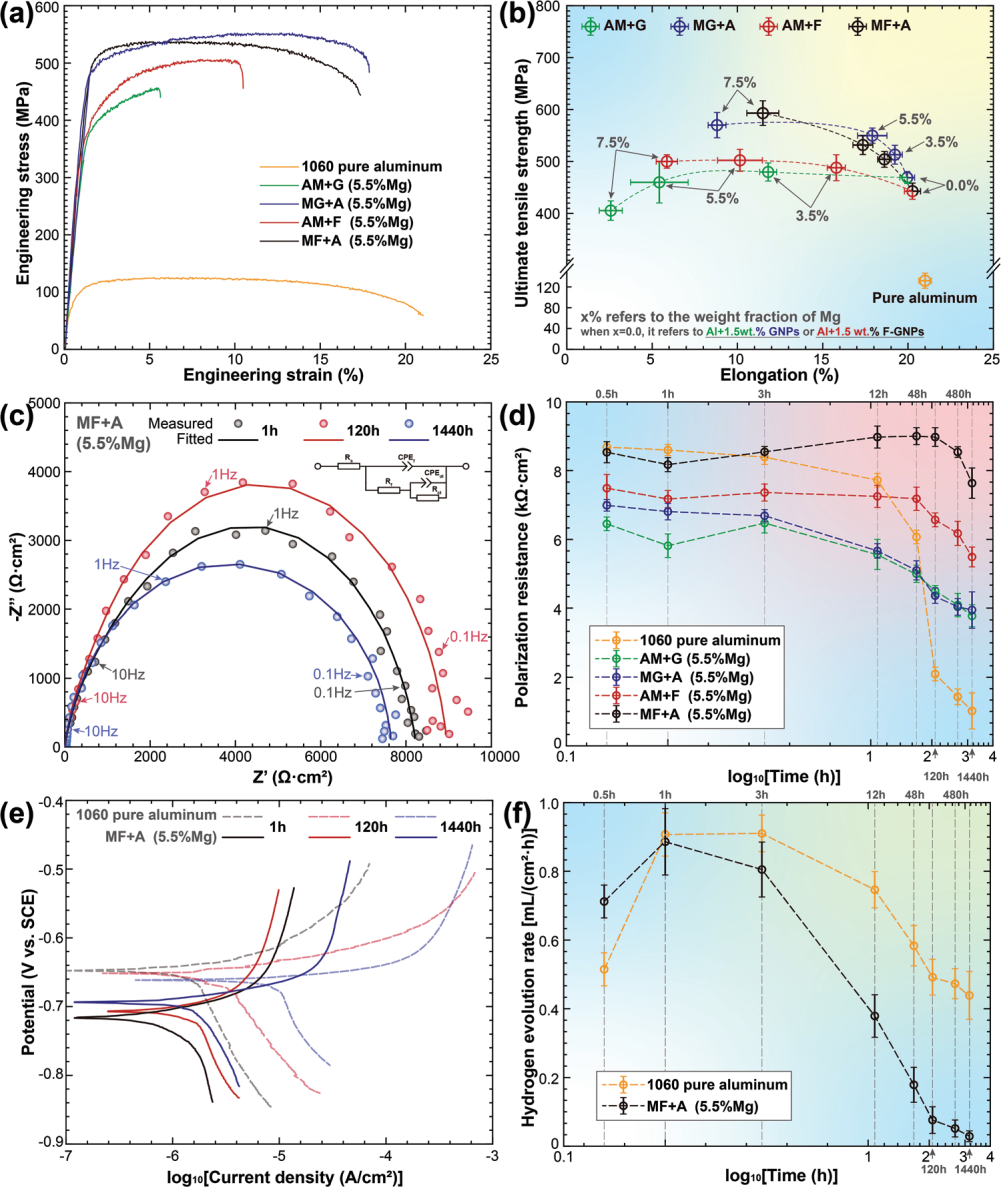
Mechanical performances and electrochemical responses of DDM composites: a) engineering stress–strain curves of composites alloyed with 5.5% Mg, b) comparison among pure aluminum and DDM composites alloyed with different routes and additions, c) EIS Nyquist spectra with different exposure time, d) polarization resistance comparison among pure aluminum and DDM composites with different routes and exposure time, e) Tafel curves extracted from PDP tests with different exposure time, and f) hydrogen evolution rate versus exposure time of pure aluminum and MF+A DDM composites.

Since our strategy is to conquer the antagonism between strength and corrosion resistance in graphene‐reinforced aluminum matrix composites, we tested the mechanical performances of the DDM composites with four different preparation routes. **Figure** **4**a shows the mechanical properties of the composites with specific Mg addition (5.5 wt%). The ball milling sequences show significant influence on the mechanical response. The strength and ductility of the composites obtained by the ball milling route of adding (F)‐GNPs firstly are significantly higher than the other two strategies. The tensile strength and elongation of the materials obtained via MG+A and MF+A reached 549 ± 32 MPa/17.9 ± 1.8% and 532 ± 39 MPa/17.3 ± 1.2%, respectively, indicating that GNPs and F‐GNPs play a very close strengthening effect. This shows that although F‐GNPs are relatively chemically inert, they still have a sound metallurgical bond with the Al matrix with the help of the Mg alloying strategy. With the load transfer effect brought by the superior specific surface area of the two‐dimensional carbonaceous nanomaterials,^[^ ^25^ ^]^ the precipitation/solid solution strengthening produced by the Mg element,^[^ ^26^ ^]^ the sound interfacial bonding between the matrix and the reinforcements,^[^ ^27^ ^]^ and the Hall‐Petch strengthening^[^ ^28^ ^]^ brought by the severe plastic deformation, the DDM composites we prepared has obtained the extraordinary mechanical performances. We further studied the comprehensive mechanical responses with different Mg addition. One can see that the tensile strength performance with all the compositions is several times that of pure aluminum, which confirms the good mechanical properties. According to our previous research,^[^ ^29^ ^]^ the comprehensive mechanical performance has reached the highest known level in related carbonaceous nanomaterial‐reinforced aluminum matrix composites. In the two preferred preparation routes, the mechanical properties of the composites basically increase gradually with the increase in the Mg addition. Regrettably, the elongation has shown a downward trend. Since ductility is also the primary component of the comprehensive mechanical properties,^[^ ^22^ ^]^ we need to strictly consider the balance of strength and ductility. As such, a 5.5% Mg addition can achieve the relatively ideal mechanical responses.

### 2.3 Electrochemical Corrosion Behaviors

Figure 4c shows the electrochemical impedance spectroscopy (EIS) response of the MF+A DDM composite versus exposure time to the 3.5 wt% NaCl aqueous solution. A capacitive loop can be seen with similar characteristics in each curve, which is caused by the overlying of two electrochemical processes. The first is caused by the charge transfer of Al to Al^3+^ at the electrical double layer formed at the corrosion surface, while the second is the relaxation and exfoliation of the corrosion products at the exposed surface.^[^ ^30^ ^]^ The passive oxide film of pure aluminum is porous in the chlorine‐containing solution, resulting in low electrical resistance. The two response loops of pure aluminum are prone to be separated.^[^ ^31^ ^]^ In contrast, due to the dense protective film formed by the (F)‐GNPs and heteroatom‐doped oxide film according to our previous research,^[^ ^32^ ^]^ the electrochemical responses in this paper cannot be distinguished, indicating that the impedance value of the doped oxide film is relatively high and shows great protection against corrosion. Besides, the larger diameter of the capacitive loop means a bigger electrochemical impedance. The corrosion resistance of MF+A DDM composites gradually increases and decreases over time. It is worth noting that even when the exposure time reaches 1440 h, that is, 2 months later, the diameter of the capacitive loop still does not show a sharp weakening trend, which shows that our composites can show good anti‐corrosion performance under long‐term service conditions. We further conducted EIS tests on various preparation routes, as well as pure aluminum, and fitted them according to the equivalent circuit in Figure 4c. Figure 4d shows the polarization resistance change extracted versus exposure time. The corrosion resistance of pure aluminum decreases drastically over time. Its initial value is close to or higher than the initial value of the DDM composites. But after 2 months of immersion, its polarization resistance was only less than 1/8 of the initial value. This shows that the oxide layer on the surface of pure aluminum can no longer maintain corrosion resistance due to the ingress of chloride ions. In contrast, the corrosion resistance of DDM composites obtained via any preparation route has only slightly decreased. Especially for the preferred preparation route MF+A, its 2‐month final state polarization resistance is about 89% of the initial state, which can be considered that there is no significant drop compared to the initial state.

Figure 4e shows the results of potentiodynamic polarization (PDP) of the MF+A DDM composites versus exposure time to the 3.5 wt% NaCl aqueous solution. Although the corrosion potentials of the composites are lower than those of the pure aluminum (this is because the equilibrium potential of Mg is lower than that of Al^[^ ^33^ ^]^), the corrosion current densities are significantly lower than that of pure aluminum. The Tafel curves only show a slight change over time, indicating that the corrosion rate is not sensitive to time. Figure 4f shows the hydrogen evolution rate of the composite materials and pure aluminum in the EXCO aqueous solution. During the first hour of exposure, both can be seen that the oxide film naturally formed on the surface gradually broke, and the hydrogen evolution process is accelerated. With the further increased time, the corrosion products begin to accumulate on the exposed surface. The diffusion distance required for the acidic chloride‐containing corrosion medium to react with the surface of the composites gradually increases, which is manifested as the gradual decrease in the rate of hydrogen evolution. In addition to the initial hydrogen evolution rate of the DDM composite is slightly higher than that of pure aluminum, while the subsequent evolution rate is significantly lower than that of pure aluminum. Especially when the time reaches 2 months, the hydrogen evolution rate is even less than 5% of pure aluminum, indicating that Mg‐alloyed F‐GNPs have a significant CSA effect. It can be presumed that the initial corrosion of the aluminum matrix contributes to the formation of a dense chemical bond between the exposed Mg‐alloyed F‐GNPs and the surface oxide film. Due to the chemical inertness of F‐GNP and its in‐plane impermeability to nearly all molecules, the diffusion distance of protons and aluminum ions in the corrosion interface is greatly increased, thereby reducing its corrosion rate.

### 2.4 Corrosion Suppression Activity

**Figure 5:**
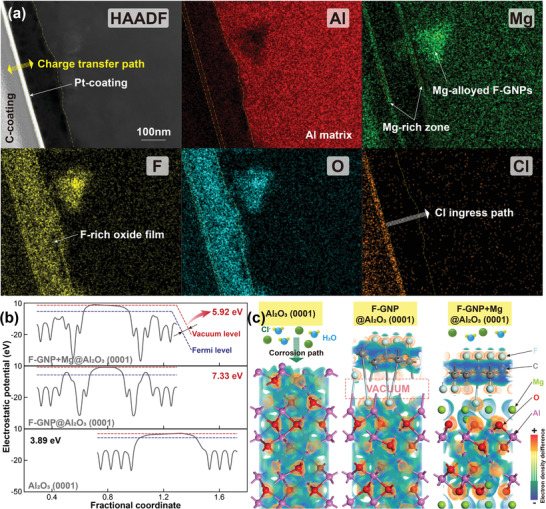
Experimental observation and theoretical calculation of the corrosion suppression activity: a) elemental distribution of the corroded surface, b) work functions, and c) electron density differences of three slabs.

Electrochemical results and hydrogen evolution rate measurements seem to prove that the corrosion resistance of the DDM composites is higher than that of pure aluminum. However, according to Sun et al.,^[^ ^34^ ^]^ undesirable anti‐corrosion weakening is easily triggered due to the CPA effect, high‐density graphene defects, and agglomeration. The problem of agglomeration can be well solved by the severe plastic deformation process.^[^ ^35^ ^]^ Our bottom‐up DDM process can realize homogeneous distribution of (F)‐GNPs in the matrix and even further reduce the thickness of graphene through interlayer shear to improve the service performance of graphene. Nevertheless, how the corrosion acceleration caused by the CPA effect and high‐density defects can be avoided or even achieved the CSA effect is still puzzling. We therefore performed elemental distribution analysis and characterization of the electrochemical corroded surface, as shown in **Figure** **5**a. The sample was prepared along the depth direction of the corroded surface via focused ion beam technique. Taking the Pt coating as the boundary, the corroded surface is shown in the right side. There is a continuous protective layer composed of dense F‐GNPs and an oxide film doped with F elements on the surface. There is also two layers of Mg as an alloying element on both sides of the protective layer. This dense protective layer plays an effective in corrosion protection. Due to the inertness of fluoride, this protective layer is theoretically unable to produce a strong metallurgical bond with the matrix. However, the presence of Mg makes up for this serious issue. Its good double‐sided metallurgical compatibility provides a strong interfacial bonding force and avoids the exfoliation of the protective layer. This continuous and dense film avoids the direct three‐phase interface among the matrix, graphene, and the corrosion medium to suppress the CPA effect. In addition, due to the honeycomb structure, the F‐GNPs form a dense delocalized cloud to block the gap within the center of its aromatic rings, which contributes to a strong repelling field again the ingress of molecules. It is well accepted that the impermeability through in‐plane graphene.^[^ ^36^ ^]^ At the junction between F‐GNPs, due to the existence of the F‐doped oxide film, these F‐GNPs form a tight bond with the oxide film, thereby avoiding the problem of the decrease in the diffusion distance of the corrosive medium caused by the microscopic defects. In a word, this layer composed of F‐GNPs and F‐doped oxide film and coated by Mg alloying finally realizes the CSA effect with a low corrosion rate better than pure aluminum over long‐term service.

To further prove the reliability of this phenomenon, we performed density function theory (DFT) calculation to clarify the electron transfer characteristics of the protective layer containing Mg‐alloyed F‐GNPs. The detailed calculation method can be found in Supplementary Information SI‐2. Figure 5b shows the work function of the Al_2_O_3_ (0001) slab, the F‐GNP@Al_2_O_3_ (0001) slab, and the F‐GNP+Mg@Al_2_O_3_ (0001) slab, respectively. The existence of F‐GNP significantly improves the work function of the composites, indicating that the difficulty of electrons removed from the matrix is increased.^[^ ^37^ ^]^ Moreover, although F‐GNP without Mg alloying seems to have a higher work function, we can see from the electron density difference image (Figure 5c) that there is a larger vacuum area between the surficial F‐GNP and the matrix. This proves that an effective chemical bond cannot be formed directly between F‐GNP and the oxide film. Due to the addition of Mg with higher electronegativity, the vacuum slab area between F‐GNP and the substrate is eliminated, which proves the sound bonding of the interface. Combined with a higher work function and a dense and homogeneous protective layer, we finally obtained breakthrough results of DDM composites with excellent corrosion performance. To our best knowledge, this is the first time to obtain the carbonaceous nanomaterial‐reinforced aluminum matrix composite with both high mechanical performances and corrosion resistance. As such, this economical bottom‐up strategy of microstructural modification broadens the horizon for anti‐corrosion engineering in aluminum matrix composites.

## 3 Conclusion

We designed a novel bottom‐up strategy to achieve charming carbonaceous nanomaterial‐reinforced aluminum matrix composites with a better balance between corrosion resistance and mechanical performances. This strategy is to conquer the corrosion promotion activity caused by heterogeneous corrosion potential, graphene defects, and agglomeration. Mg‐alloyed fluorinated graphene nanoplatelets were introduced via severe plastic deformation and designed preparation routes. Homogeneously dispersed Mg‐alloyed fluorinated graphene nanoplatelets and ultrafine‐grained microstructures contributed to the formation of dense and strong protective film again chloride‐containing aqueous environment. Corrosion suppression activity was discovered in the designed composites. The anti‐corrosion polarization resistance remained 89% of the initial state after 2‐month immersion in chloride‐containing environment, while the ultra‐tensile strength and elongation of 532 ± 39 MPa and 17.3 ± 1.2% were achieved. The microstructural characterization and density function theory calculation confirmed the existence and rationality of the dense anti‐corrosion layer composed of fluorinated graphene nanoplatelets and F‐doped oxide film and coated by Mg alloying. Moreover, this work is significant for conquering the antagonism between mechanical performances and corrosion resistance, which is indeed critical for the design of carbonaceous nanomaterial‐reinforced aluminum matrix composites to realize some desired performance for practical applications.

## Conflict of Interest

The authors declare no conflict of interest.

## Supporting information

Supporting Information

## Data Availability

The data that support the findings of this study are available in the supplementary material of this article.
