# Supplementary material for: Heteroatom Modification Enhances Corrosion Durability in High‐Mechanical‐Performance Graphene‐Reinforced Aluminum Matrix Composites
Source: Adv Sci (Weinh). 2022 Jun 15;9(23):2104464. doi: 10.1002/advs.202104464 (PMC9376822; doi:10.1002/advs.202104464)
Supplement: Supplementary file 1 — Supporting Information [file ADVS-9-2104464-s001.pdf]

ADVANCED SCIENCE
Open Access

Supporting Information

for *Adv. Sci.*, DOI 10.1002/advs.202104464

Heteroatom Modification Enhances Corrosion Durability in High-Mechanical-Performance Graphene-Reinforced Aluminum Matrix Composites

*Yuming Xie, Xiangchen Meng, Yuexin Chang, Dongxin Mao, Zhiwei Qin, Long Wan and Yongxian Huang**

Supplementary Information

Heteroatom modification enhances corrosion durability in high-mechanical-performance graphene-reinforced aluminum matrix composites

*Yuming Xie †, Xiangchen Meng †, Yuexin Chang, Dongxin Mao, Zhiwei Qin, Long Wan, Yongxian Huang **

State Key Laboratory of Advanced Welding and Joining, Harbin Institute of Technology, 150001 Harbin, China.

† These authors contributed equally to this work.

* Corresponding author. Tel: 86-451-86413951. E-mail: yxhuang@hit.edu.cn (Yongxian Huang).

S-1. Experimental section

a) Materials and preparations

Mg-doped F-GNP reinforced aluminum matrix composites were fabricated by a bottom-up preparation route consisting of sequential ball milling and DDM. Atomized aluminum powders with an average diameter of 10 μm and a purity of 99.6% were blended with the appropriate amount of atomized magnesium powders (10 μm in diameter) and F-GNPs (XF079, Nanjing XFNANO Materials Tech Co., Ltd, China) by a planetary ball milling equipment. The addition of F-GNPs is 1.5 wt.%. Yttria stabilized zirconia ball of 6 mm (70%) and 8 mm (30%) in diameter were used, and the ball powder ratio was 10:1. Polyethylene glycol 2000 with a weight fraction of 4% was added as process control agent. The mixed powder was ball milled under argon atmosphere at a rotational velocity of 200 rpm. Two kinds of ball milling sequences were designed as shown in Fig. 1. The as-milled composite powders were sintered at 400 ℃ in argon atmosphere to remove the process control agent.

The obtained powders were cold-pressed at about 200 MPa into a pre-densified compact with a weight of 0.82±0.01 g. A DDM tool rotating at a specific rotational velocity of 1000 rpm pressed the compact for 60 seconds to introduce severe plastic deformation and corresponding heat input for the sintering of the final composite disc. The final diameter and thickness were 16 mm and 1.0 mm, respectively. For comparison, aluminum matrix composites reinforced by Mg-doped GNPs (XF022-1,

Nanjing XFNANO Materials Tech Co., Ltd, China) were also fabricated by the same route. The detailed DDM procedure could be obtained from our previous work [1–3].

b) Characterization

Field emission-scanning electron microscope (FE-SEM, HITACHI SU5000) equipped with backscatter electron (BSE) detector and energy dispersive spectroscopy (EDS) was utilized to show the dispersion and homogeneity of the microstructure and the morphologies of the corroded surfaces. The grain features of samples were characterized by the electron backscatter diffraction (EBSD). The data of EBSD results were analyzed by AZtecHKL software. The phase composition and lattice distortion of the samples was analyzed based on the X-ray diffraction (XRD, PANalytical X'PERT) data. Transmission electron microscope (TEM, FEI Talos F200x) was applied to examine the detailed microstructure among Al matrix, Mg coating, and F-GNPs with an accelerating voltage of 200 kV. Raman spectroscopy was conducted using Renishaw inVia-Reflex to evaluate the disordering and defect density of F-GNP structures X-ray photoelectron spectroscopy (XPS, ThermoFisher ESCALAB 250Xi) was utilized to obtain the chemical bonding evolution during the DDM process.

c) Corrosion measurements

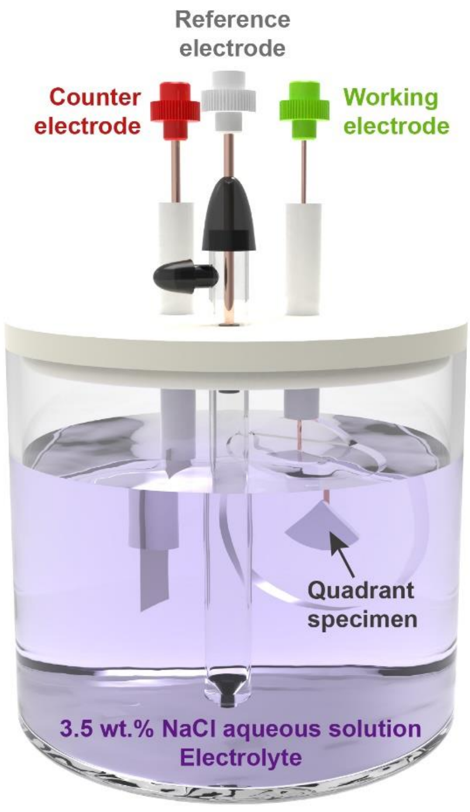

Figure S1 Schematic of the three-electrode system.

Specimens cut from Mg-coated F-GNP/GNP reinforced aluminum matrix composite disc were tested by electrochemical measurement with a quadrant shape and a diameter of 16 mm. A CHI 760E system for potentiodynamic polarization (PDP) and a PARSTAT 4000A system for electrochemical impedance spectroscopy (EIS) and Mott-Schottky analyses. A three-electrode system was used in the test and the applied electrolyte was a 3.5 wt.% NaCl aqueous solution at room temperature, as shown in Fig. S1. The cut specimens were used as working electrodes. A Pt foil and a saturated calomel electrode (SCE) were used as the counter electrode and reference electrode. A stabilization time by immersing the working electrodes into the electrolyte for one hour was used before initial PDP and EIS tests. The PDP data of each specimen were recorded by continuously scanning at a scanning rate of 50 mV/min, and each test was repeated for three times. EIS measurements were conducted at the open circuit at frequencies ranging from 1 MHz to 0.01 Hz with a sinusoidal perturbation of 5 mV in amplitude. Mott-Schottky characteristics were acquired by monitoring the frequency-potential response at 1 kHz and 10 kHz between -1.5 V to 0.0 V vs. SCE with a scanning interval of 25 mV.

The corrosion rate of these aluminum matrix composites was estimated from the continuous hydrogen evolution collections. The applied experimental procedure for hydrogen collection was shown in Fig. S2. The evolved hydrogen gas was collected for each quadrant specimen and measured by the volume of discharged deionized water. 30 days of immersion in 500 mL EXCO aqueous solution at room temperature were selected to monitor the discharged volume. Each specimen was mounted by polymethyl methacrylate with an exposed area of 0.5 cm^2 and fine ground by 7000 grit SiC paper. No renewing or agitating of the static solution was conducted during the entire measurement. The immersion tests were repeated three times for better statistics.

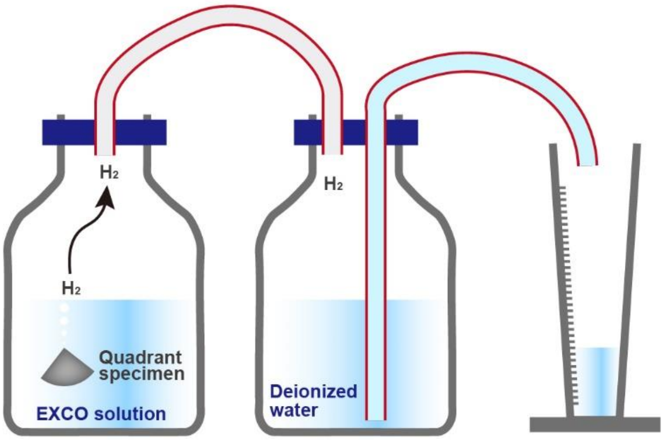

Figure S2 Schematic of the hydrogen collection system.

d) Statistical Analysis

OriginPro (Version 2021) were used for the statistical analysis of the data presented in this work. All data in Figures 3a and 4 were presented as mean ± SD (standard deviation).

S-2. Density functional theory calculation

As shown in Fig. S3, a simple α-Al_2O_3 (0001) slab with a thickness of approximately 11 Å was constructed for density functional theory (DFT) calculations. F-GNP@Al_2O_3 (0001) slab and F-GNP+Mg@Al_2O_3 (0001) slab were also established by replacing partial Al_2O_3 layer with F-GNP and Mg-alloyed F-GNP, respectively. A sufficient large vacuum of approximately 20 Å was added along the [0001] direction to avoid interaction between the neighboring slabs along Z-axis. Norm-conserving pseudopotentials were implemented via QUANTUM ESPRESSO [4] for the DFT calculation. The system of Kohn-Sham equations was self-consistently solved on the plane wave basis to determine the electron density of the system. We performed integration in reciprocal space on a $5\times5\times1$ Monkhrost-Pack lattice of k-points [5]. The threshold for the maximum kinetic energy of plane waves included in the basis set was 830 eV. Calculations were performed with the PBE exchange-correlation function in the generalized gradient approximation (GGA). These slabs were firstly relaxed until the energy, residual force and displacement were less than 5.0×10^{-6} eV/atom, 0.01 eV/Å, and 5.0×10^{-4} Å, respectively. Work function, electron density difference, and local partial density of states were calculated to illustrate the corrosion suppression activity brought by Mg-alloyed F-GNPs.

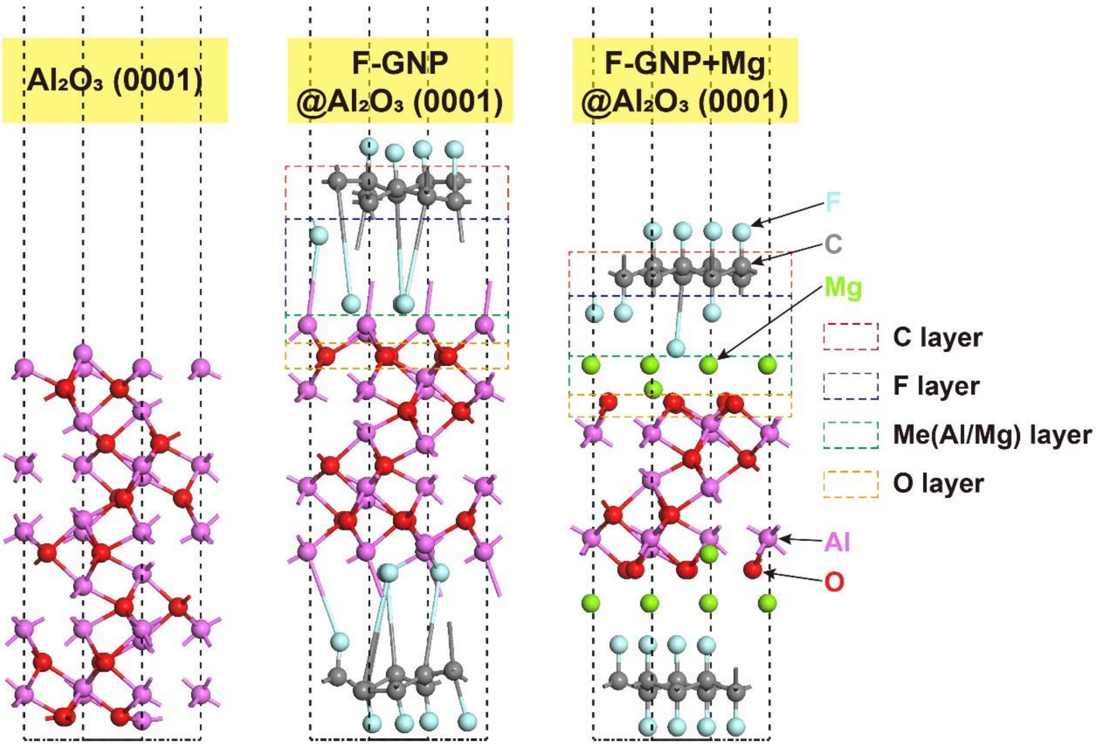

Figure S3 Schematics of Al_2O_3 (0001) slab, F-GNP@Al_2O_3 (0001) slab, and F-GNP+Mg@Al_2O_3 (0001) slab.

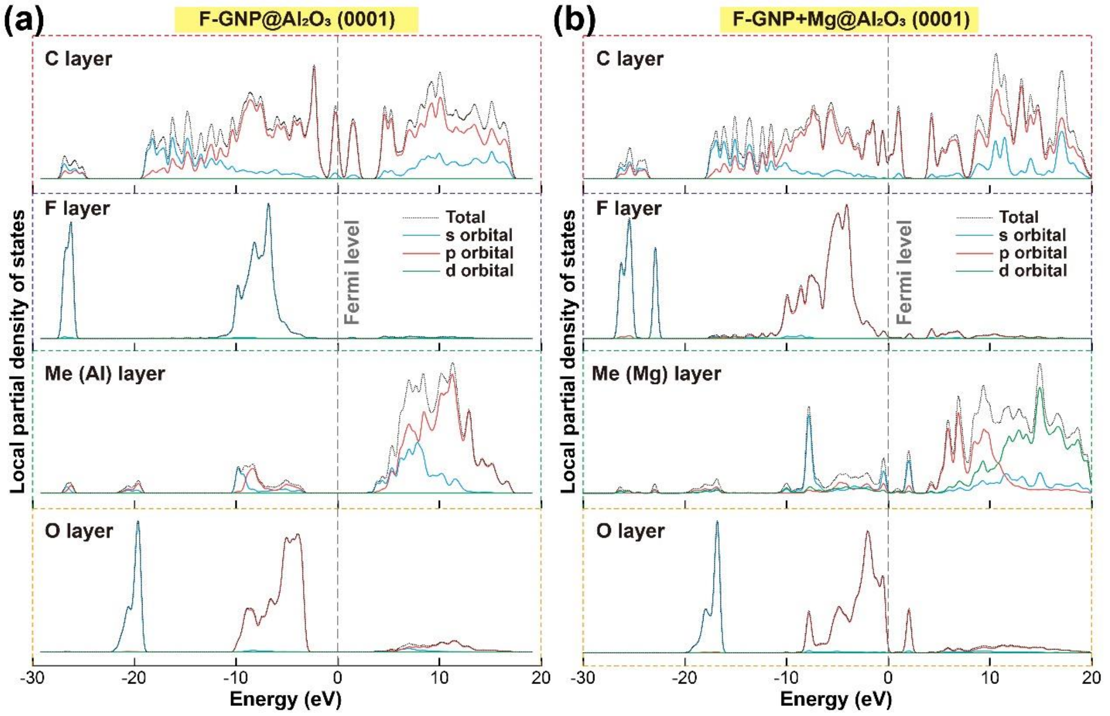

Figure S4 Local partial density of states of (a) F-GNP@Al_2O_3 (0001) slab and (b) F-GNP+Mg@Al_2O_3 (0001) slab.

S-3. Microstructural supplementary information

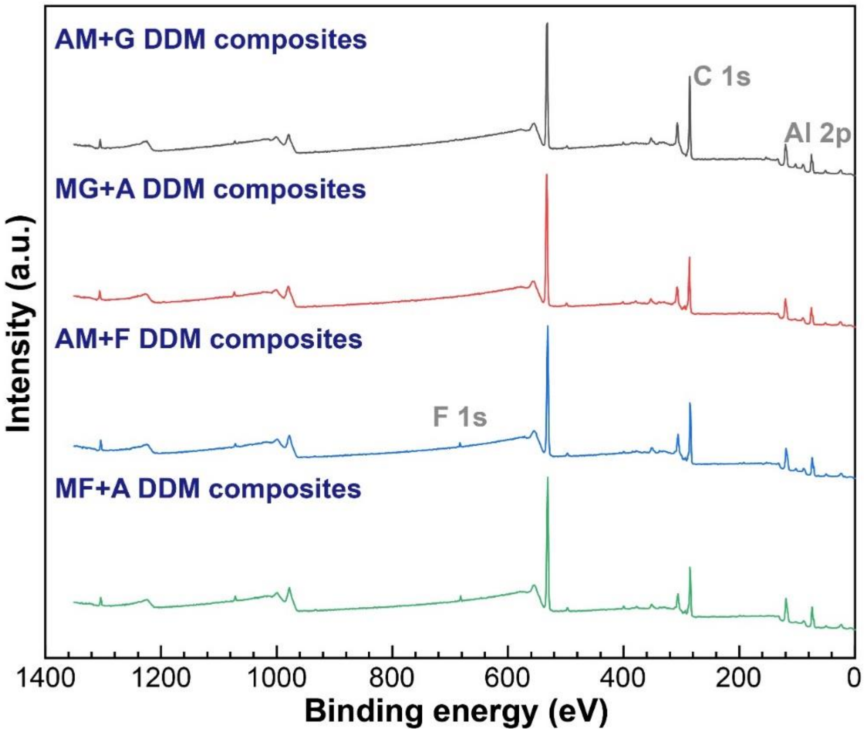

Figure S5 XPS full spectra of AM+G, MG+A, AM+F, and MF+A DDM composites.

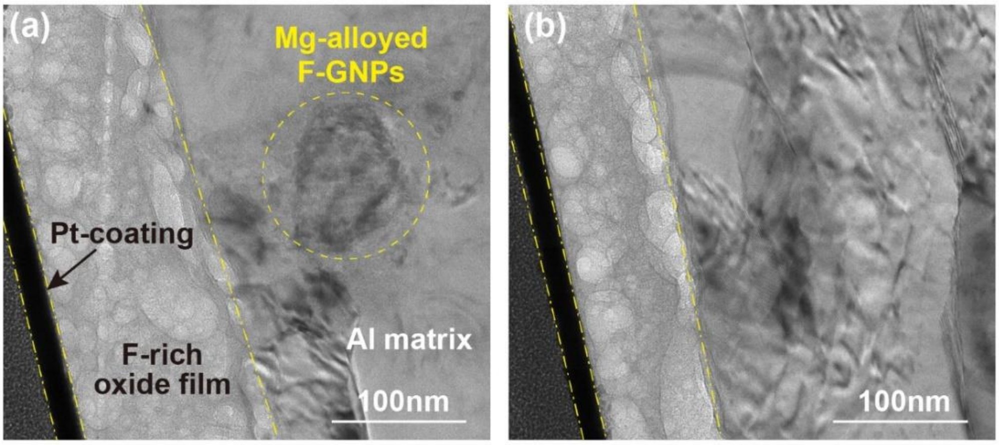

Figure S6 TEM images of corrosion surfaces via FIB techniques: (a) and (b) part of the corrosion surface.

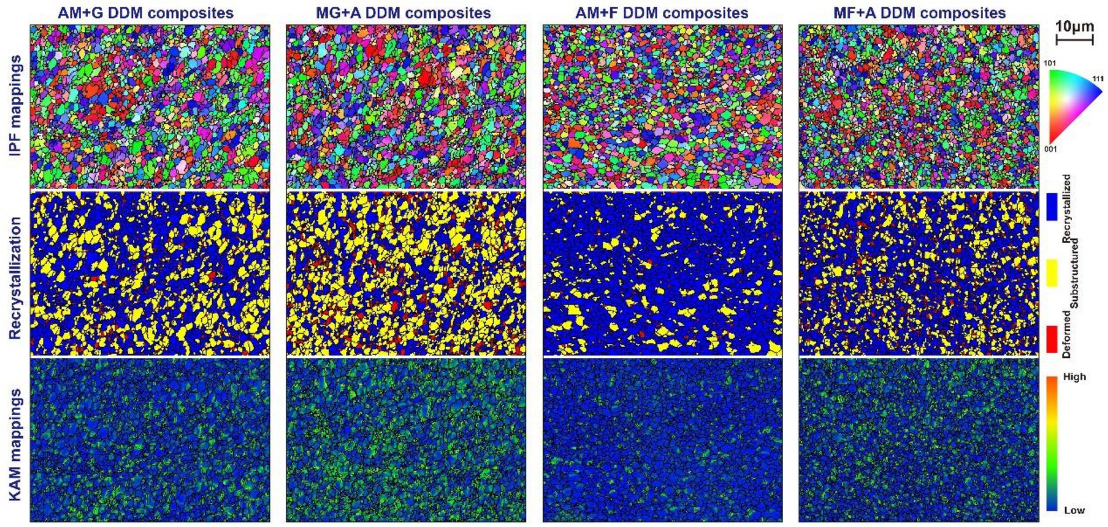

Figure S7 EBSD IPF mappings, recrystallization mappings, and kernel average misorientation (KAM) mappings of AM+G, MG+A, AM+F, and MF+A DDM composites.

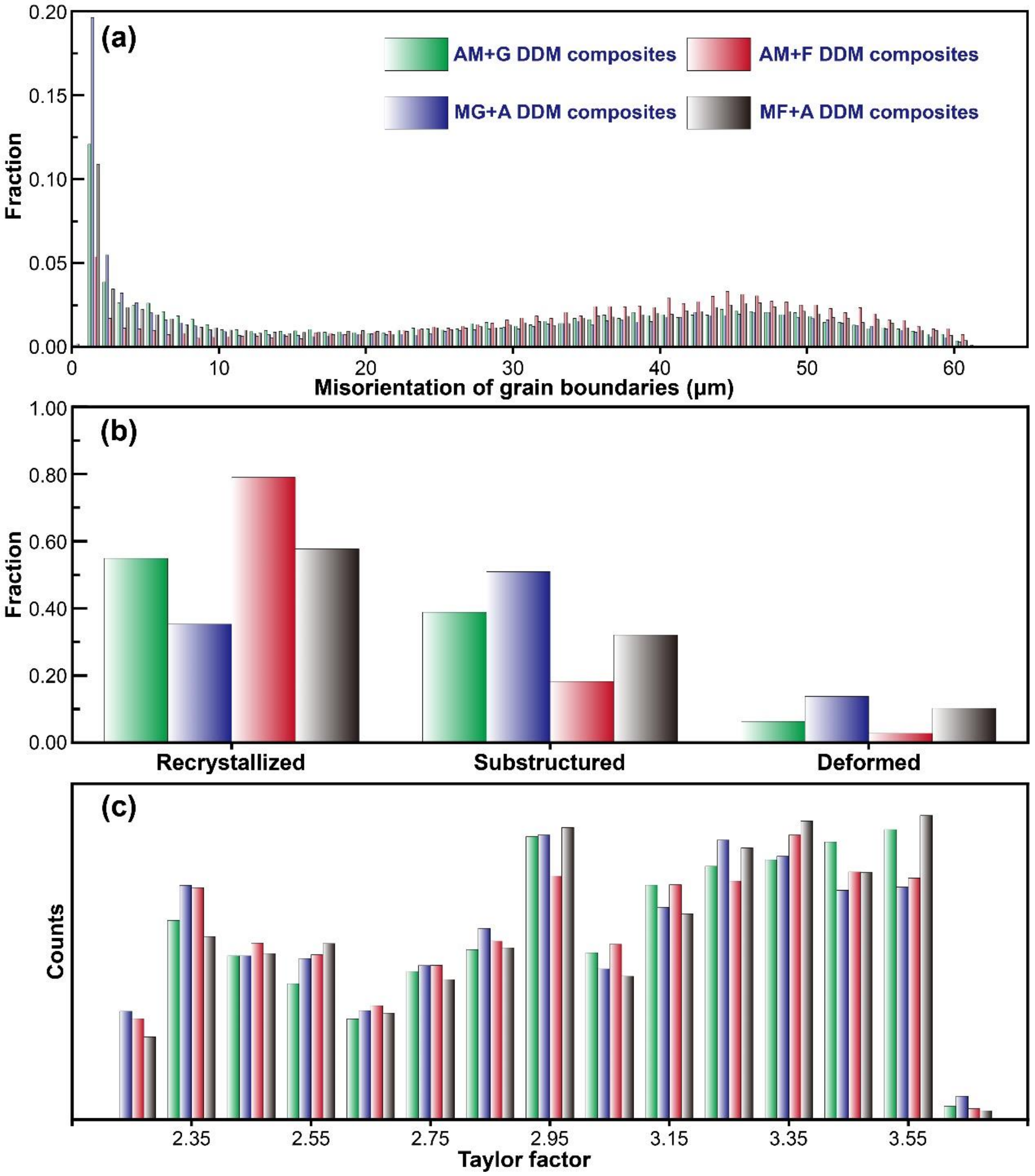

Figure S8 EBSD factors: (a) diameter distribution of grains, (b) recrystallization fraction, and (c) distribution of Taylor factors.

References

[1] Y. Xie, X. Meng, Y. Huang, J. Li, J. Cao, *Compos. Part B Eng.* **2019**, *177*, 107413.

[2] Y. Xie, X. Meng, Y. Li, D. Mao, L. Wan, Y. Huang, *Compos. Commun.* **2021**, 100776.

[3] Y. Xie, X. Meng, D. Mao, Z. Qin, L. Wan, Y. Huang, *ACS Appl. Mater. Interfaces* **2021**, acsami.1c07148.

[4] P. Giannozzi, S. Baroni, N. Bonini, M. Calandra, R. Car, C. Cavazzoni, D. Ceresoli, G. L. Chiarotti, M. Cococcioni, I. Dabo, A. Dal Corso, S. de Gironcoli, S. Fabris, G. Fratesi, R. Gebauer, U. Gerstmann, C. Gougoussis, A. Kokalj, M. Lazzeri, L. Martin-Samos, N. Marzari, F. Mauri, R. Mazzarello, S. Paolini, A. Pasquarello, L. Paulatto, C. Sbraccia, S. Scandolo, G. Sclauzero, A. P. Seitsonen, A. Smogunov, P. Umari, R. M. Wentzcovitch, *J. Phys. Condens. Matter* **2009**, *21*, 395502.

[5] M. Liu, Y. Jin, C. Leygraf, J. Pan, *J. Electrochem. Soc.* **2019**, *166*, C3124.
